# Osteopontin Deficiency Alters Biliary Homeostasis and Protects against Gallstone Formation

**DOI:** 10.1038/srep30215

**Published:** 2016-08-03

**Authors:** Jing Lin, Wei-qing Shao, Zong-you Chen, Wen-wei Zhu, Lu Lu, Duan Cai, Lun-xiu Qin, Hu-liang Jia, Ming Lu, Jin-hong Chen

**Affiliations:** 1 Department of General Surgery, Huashan Hospital, Fudan University, Shanghai, 200040, China

## Abstract

The precipitation of excess biliary cholesterol as solid crystals is a prerequisite for cholesterol gallstone formation, which occurs due to disturbed biliary homeostasis. Biliary homeostasis is regulated by an elaborate network of genes in hepatocytes. If unmanaged, the cholesterol crystals will aggregate, fuse and form gallstones. We have previously observed that the levels of osteopontin (OPN) in bile and gallbladder were reduced in gallstone patients. However, the role and mechanism for hepatic OPN in cholesterol gallstone formation is undetermined. In this study, we found that the expression of hepatic OPN was increased in gallstone patients compared with gallstone-free counterparts. Then, we observed that OPN-deficient mice were less vulnerable to cholesterol gallstone formation than wild type mice. Further mechanistic studies revealed that this protective effect was associated with alterations of bile composition and was caused by the increased hepatic CYP7A1 expression and the reduced expression of hepatic SHP, ATP8B1, SR-B1 and SREBP-2. Finally, the correlations between the expression of hepatic OPN and the expression of these hepatic genes were validated in gallstone patients. Taken together, our findings reveal that hepatic OPN contributes to cholesterol gallstone formation by regulating biliary metabolism and might be developed as a therapeutic target for gallstone treatments.

Gallstone disease is a major health problem worldwide, and its associated complications and comorbidities impose a substantial financial burden on the health care economy^1^,^2^,^3^,^4^. Gallstone disease is a multifactorial disease influenced by a complex interaction of genetic and environmental factors^5^. The precipitation of excess cholesterol in bile as solid crystals is a prerequisite for cholesterol gallstone formation^6^,^7^. Additionally, some biliary proteins, namely pro-nucleation and anti-nucleation proteins, could also influence cholesterol crystals and stone formation. The critical balance between these proteins determines the predisposition of bile to form cholesterol crystals or prolong the process of crystal formation^8^. The solubility of cholesterol in aqueous solutions is extremely limited. However, cholesterol could be made soluble in bile through mixed micelles composed of bile salts and phospholipid^5^. Cholesterol precipitation results from excessive cholesterol, deficiency in bile salts or phospholipid, or a combination of these factors^5^. The metabolism of bile salts and lipids is regulated by an elaborate network of transporters. Briefly, cholesterol secretion is regulated by the ABC binding cassette (ABC) transporters ABCG5, ABCG8 and Scavenger receptor class, B1 (SR-B1)^9^,^10^,^11^. The secretion of phospholipid is controlled by ABCB4, a P-glycoprotein member of the multi-drug resistance gene family^12^. Then, bile acids are secreted into the bile by ABCB11 and ABCB1a/b^13^. If the function of these transporters is disturbed, resulting in unbalanced biliary homeostasis, the cholesterol crystals will aggregate, fuse, and ultimately form pathologic gallstones.

Osteopontin (OPN) is a soluble cytokine and a matrix-associated protein expressed in the majority of tissues and body fluids^14^ and is able to control tumour progression and metastasis^15^. Our previous studies demonstrated that OPN can inhibit cholesterol gallstone formation as an anti-nucleation factor in gallbladder bile^16^,^17^. Another study showed that OPN was highly expressed in the epithelium of stone-laden intrahepatic bile ducts, intramural, extramural glands and stones, indicating that OPN is involved in hepatolithiasis^18^. However, the role of hepatic OPN in cholesterol gallstone formation is undetermined. Chapman J. *et al*. found that OPN-deficient (OPN−/−) mice were completely protected from hepatic insulin resistance which developed in wild type (WT) controls when fed a high-fat diet for 2–4 weeks^19^. Biddinger S.B. *et al*. observed that hepatic insulin resistance directly promoted the formation of cholesterol gallstones by increasing the expression of the biliary cholesterol transporters ABCG5 and ABCG8 and decreasing that of the bile acid synthetic enzymes in mice^20^. These studies suggest that OPN may regulate hepatic bile salts and lipid metabolism and affect cholesterol gallstone formation.

In this study, we analysed the correlation between hepatic OPN expression and gallstone formation both in patients and in mice. We reveal that hepatic OPN contributes to cholesterol gallstone formation by regulating biliary metabolism in mice.

## Results

### Clinical characteristics and hepatic expression of OPN in gallstone patients (GS) and gallstone-free patients (GSF)

**Figure 1:**
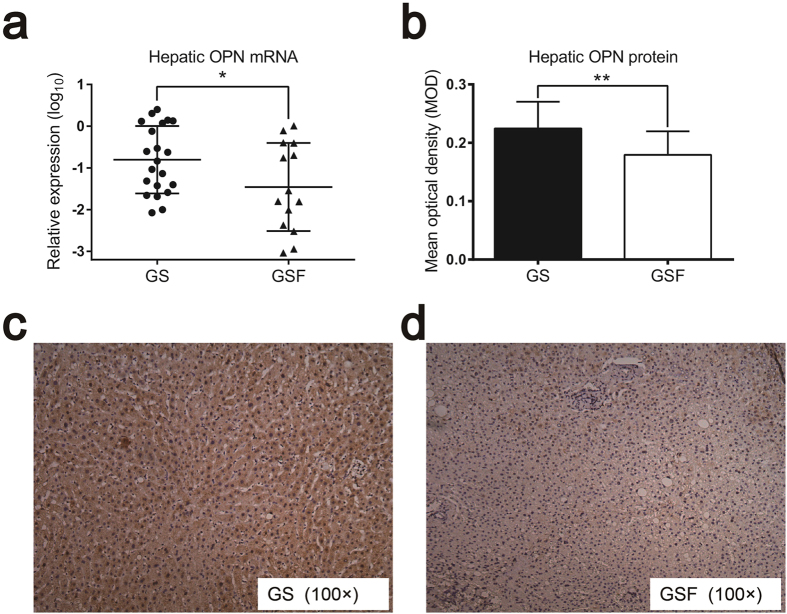
Expression of hepatic OPN in gallstone patients (GS) and gallstone-free patients (GSF). (**a**) Quantitative real-time PCR analysis of hepatic OPN mRNA levels in the liver tissues of GS and GSF. The mRNA expression of hepatic OPN was log-transformed, prior to analysis. The mRNA expression of hepatic OPN was higher in GS than in GSF. **(b)** The OPN immunohistochemical mean optical density (MOD) differed significantly between the GS and female GSF. The data are expressed as the mean ± SD (GS: n = 21, GSF: n = 14). **(c)** Hepatic specimens shown are from GS that were immunohistochemically stained for OPN (magnification, ×100). **(d)** Hepatic specimens shown are from GSF that were immunohistochemically stained for OPN (magnification, ×100). Statistical analysis was performed using unpaired Student’s t test, *P < 0.05, **P < 0.01.

To investigate the role of hepatic OPN in gallstone formation, we first analysed the expression of OPN in liver tissue samples of GS and GSF by quantitative real-time PCR. The messenger RNA (mRNA) expression of hepatic OPN was higher in GS than in GSF (Fig. 1a). The results from quantitative immunohistochemistry also showed that the protein expression of hepatic OPN was increased in GS (Fig. 1b–d). No significant difference in age, gender, body mass index or fasting glucose was observed between the GS and GSF groups (Supplementary Table S1). These results suggest that hepatic OPN plays an important role in the formation of pathologic gallstones.

### OPN deficiency reduces diet-induced cholesterol gallstone formation in mice

**Figure 2:**
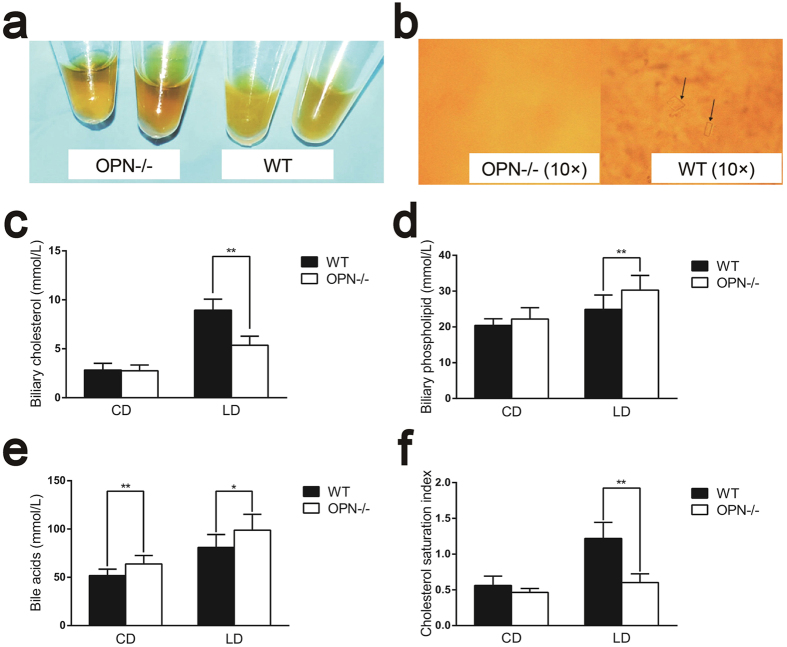
Loss of OPN alters biliary compositions and protects against gallstone formation in mice. Bile was harvested from WT and OPN−/− mice. **(a)** Gross appearance of representative bile from WT and OPN−/− mice fed a LD. **(b)** Polarizing light microscopic examination of biliary cholesterol crystals (indicated by black arrows) from WT and OPN−/− mice fed a LD (magnification, ×10). **(c–f)** The biliary cholesterol contents **(c)**, phospholipid levels **(d)**, bile acid contents **(e)** and CSI **(f)** were measured in OPN−/− mice and WT mice fed a CD or LD. The data are expressed as the mean ± SD (CD: n = 9, LD: n = 10). Statistical analysis was performed using unpaired Student’s t test, *P < 0.05, **P < 0.01. WT, wild type; OPN−/−, OPN deficient; CD, chow diet, LD, lithogenic diet; CSI, cholesterol saturation index.

Next, OPN−/− mice were used to further investigate the role and mechanism of hepatic OPN in gallstone formation. When fed a chow diet (CD), neither WT mice nor OPN−/− mice showed crystals or gallstones (Table 1). Eighty per cent (4 of 5 males and 4 of 5 females) of WT mice developed gallstones when fed a lithogenic diet (LD) for 8 weeks whereas the penetrance in OPN−/− mice was 10% (1 of 5 males and 0 of 5 females) (Table 1). Compared with that of OPN−/− mice, the gallbladder bile of WT mice appeared turbid and full of precipitates and stones (Fig. 2a). Microscopic examination of the gallbladder bile revealed cholesterol crystals in WT mice, whereas OPN−/− mice were largely free of cholesterol precipitates (Fig. 2b). Gallbladder and liver histology was similar in mice fed a LD (Supplementary Fig. S1).

### OPN deficiency alters the biliary composition and cholesterol saturation index (CSI) in mice

OPN−/− mice fed a CD exhibited increased bile acid levels (Fig. 2e, left column) but similar biliary cholesterol and phospholipid levels compared with WT mice (Fig. 2c,d, left columns). However, OPN−/− mice fed a LD for 8 weeks showed an increase in phospholipid and bile acid levels (Fig. 2d,e, right columns) but a reduction in cholesterol content compared with WT mice (Fig. 2c, right column). This combined effect of biochemical alterations led to a decrease of the CSI in LD-fed OPN−/− mice (Fig. 2f, right columns), providing a biochemical mechanism for OPN deficiency in protecting mice against cholesterol gallstone formation.

### OPN deficiency alters the body weight, liver weight and hepatic lipid and bile acid contents but not the serum lipid and bile acid profiles in mice

There was no difference in body weight, liver weight, or the lipid and bile acid profiles of liver and serum between the WT mice and OPN−/− mice fed a CD (Table 2). After 8 weeks of being fed a LD, OPN−/− mice showed an increase in body weight but a decrease in their liver weight/body weight ratio compared with WT mice (Table 2). The serum lipid profile between the two genotypes presented no change after 8 weeks of LD. Though the serum bile acid tended to be increased in OPN−/− mice fed a LD, the change was not statistically significant (Table 2). The hepatic lipid contents, including cholesterol and triglyceride (TG), and hepatic bile acid levels were higher in OPN−/− mice than in WT mice fed a LD (Table 2). These findings suggest that LD is accompanied by distinctive alterations in hepatic lipid and bile acid metabolism in OPN−/− mice compared with WT mice.

### OPN deficiency alters the expression of hepatic genes involved in cholesterol and bile acid metabolism

**Figure 3:**
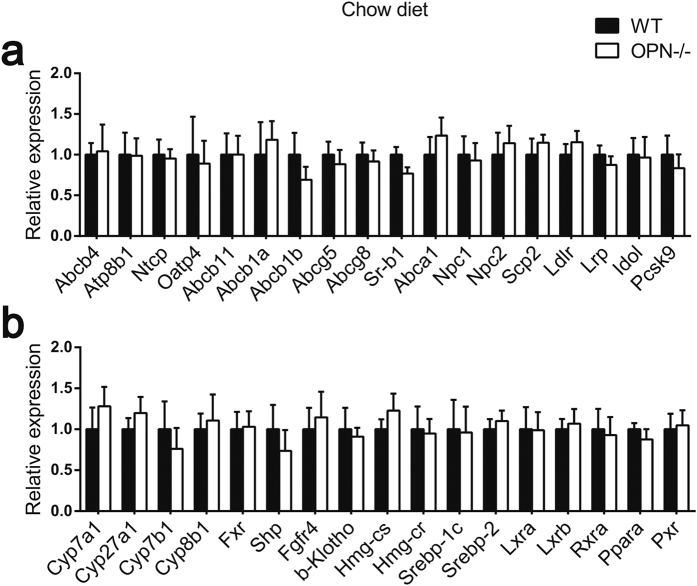
Expression of cholesterol and bile acid metabolism-related genes in mice fed a chow diet. Quantitative real-time PCR analysis of mRNA levels for genes involved in cholesterol metabolism and bile acid metabolism in liver tissues from WT mice and OPN−/− mice fed a chow diet. The data are expressed as the mean ± SD (n = 8 per group). Statistical analysis was performed using unpaired Student’s t test, *P < 0.05, **P < 0.01. WT, wild type; OPN−/−, OPN deficient.

**Figure 4:**
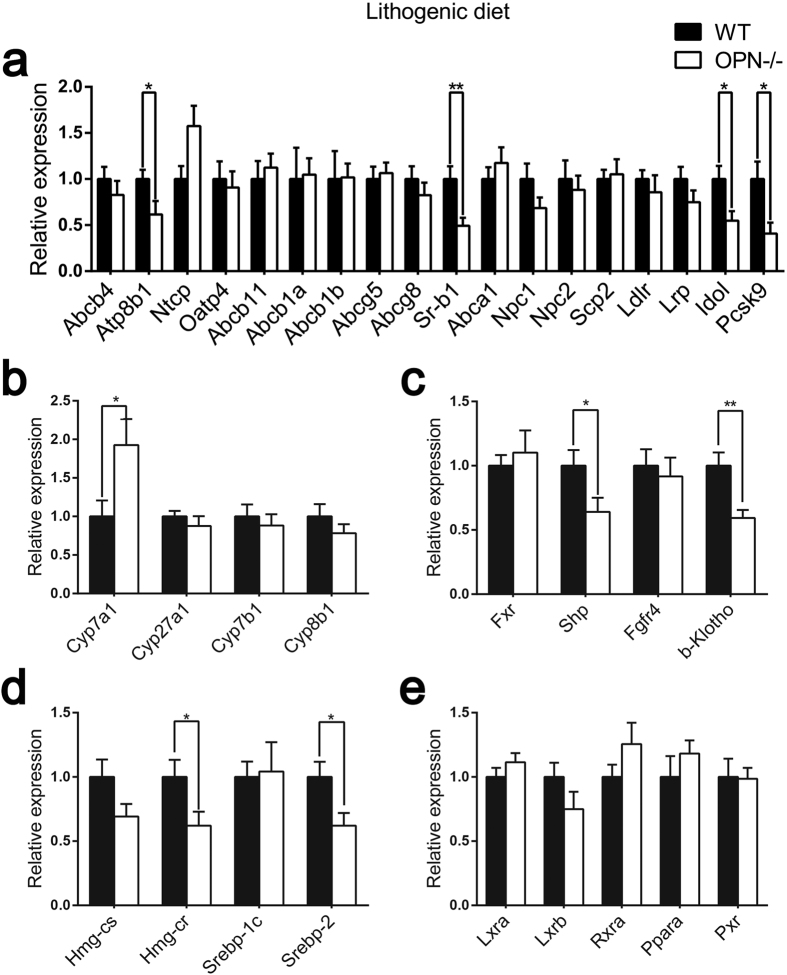
Expression of cholesterol and bile acid metabolism-related genes in mice fed a lithogenic diet. Quantitative real-time PCR analysis of mRNA levels for genes involved in cholesterol metabolism and bile acid metabolism in liver tissues from WT mice and OPN−/− mice fed a lithogenic diet. The data are expressed as the mean ± SD (n = 8 per group). Statistical analysis was performed using unpaired Student’s t test, *P < 0.05, **P < 0.01. WT, wild type; OPN−/−, OPN deficient.

To understand the mechanism by which OPN deficiency protects mice from cholesterol gallstone formation, we analysed the expression of hepatic genes that are involved in biliary homeostasis. There were no significant differences in those genes between the two genotypes fed a CD (Fig. 3a,b). When challenged with the LD for 8 weeks, we first measured the expression of hepatic bile acid and lipid transporters. The expression of the phospholipid reverse transporters, ATPase, aminophospholipid transporter, class I, type 8B, member 1 (ATP8B1) was reduced by almost 40% in OPN−/− mice whereas the expression of phospholipid efflux ABCB4 was unchanged (Fig. 4a). Among bile acid transporters, OPN−/− mice showed no difference in the expression of Na^+^ taurocholate cotransporting polypeptide (NTCP), organic anion transporter (OATP), ABCB11 or ABCB1a/b (Fig. 4a). Among the cholesterol transporters, the expression of SR-B1 was decreased by almost 50% in OPN−/− mice whereas the expression of ABCG5, ABCG8 and ABCA1 was not affected (Fig. 4a). The expression of the intracellular cholesterol transporters Niemann pick type C1 (NPC1), Niemann pick type C2 (NPC2) and sterol carrier protein 2 (SCP2) showed no difference (Fig. 4a). The expression of low density lipoprotein receptor (LDLR) and LDLR-related protein (LRP) was also unchanged, whereas the expression of inducible degrader of the low-density lipoprotein receptor, LDLR (IDOL) and proprotein convertase subtilisin/kexin type 9 (PCSK9) was significantly reduced in OPN−/− mice fed a LD (Fig. 4a).

The biosynthesis of bile acids in the liver is controlled by multiple CYP enzymes. The mRNA level of cholesterol 7-alpha-monooxygenase (CYP7A1) was markedly increased in LD-fed OPN−/− mice. However, the expression of other CYP enzymes was not significantly changed (Fig. 4b). The expression of the CYP7A1 activator liver X receptor (LXR) showed no difference (Fig. 4e). We then evaluated the expression of farnesoid X receptor (FXR), small heterodimer partner (SHP), fibroblast growth factor receptor 4 (FGFR4) and beta-klotho (β-KLOTHO), which play important roles in the negative feedback control of CYP7A1 transcription. The expression of SHP and β-KLOTHO was decreased in OPN−/− mice whereas the expression of FXR and FGFR4 was not affected (Fig. 4c).

**Figure 5:**
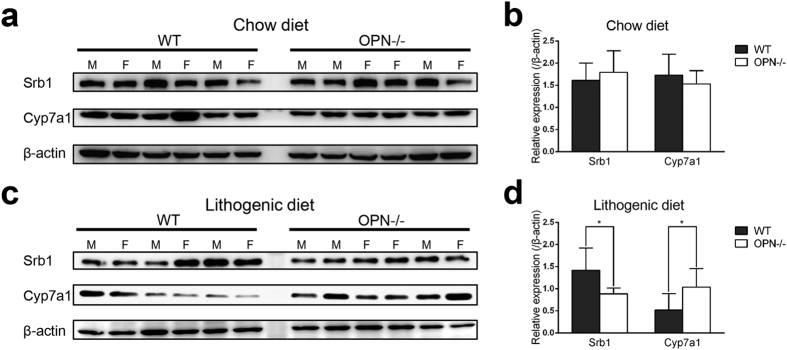
Western blot analysis of expression of SR-B1 and CYP7A1 in mice. (**a**,**c**) Hepatic proteins were isolated from WT and OPN−/− mice and analysed by Western blotting for SR-B1 and CYP7A1 protein expression. The gender of mice was labelled above the bands. β-Actin was selected as a control for gel loading. **(b,d)** Quantification of Western blots. The relative average protein level was determined by densitometry. The data are expressed as the mean ± SD (n = 6 per group, 3 female and 3 male mice). Statistical analysis was performed using unpaired Student’s t test, *P < 0.05, **P < 0.01. WT, wild type; OPN−/−, OPN deficient; M, male; F, female.

We next evaluated the expression of 3-hydroxy-3-methylglutaryl coenzyme A synthase (HMGCS) and 3-hydroxy-3-methylglutaryl coenzyme A reductase (HMGCR), the two key enzymes in hepatic cholesterol synthesis. The expression of HMGCR was reduced by 40% in OPN−/− mice whereas the expression of HMGCS showed no difference. We then measured two upstream transcriptional factors, sterol regulatory element binding protein 1c (SREBP-1c) and sterol regulatory element binding protein 2 (SREBP-2). The expression of SREBP-2 was reduced in OPN−/− mice whereas the expression of SREBP-1c was not affected (Fig. 4d). We also profiled the expression of other nuclear receptors that may affect the biliary homeostasis, but they showed no significant change (Fig. 4e). The change in expression of SR-B1 and CYP7A1 protein was confirmed by Western blot analysis (Fig. 5).

**Figure 6:**
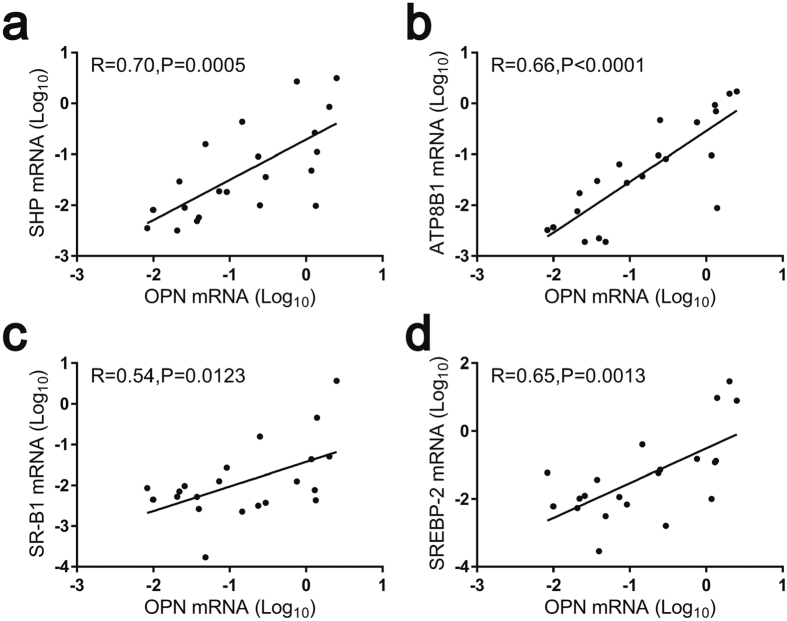
Correlations of expression of cholesterol and bile acid metabolism-related genes and the OPN gene in liver tissues of gallstone patients. Relative mRNA levels were analysed by quantitative real-time PCR. The mRNA expression of the genes was log-transformed, prior to analysis. A correlation between hepatic SHP and OPN mRNA levels (n = 21) **(a)** hepatic ATP8B1 and OPN mRNA levels (n = 21) **(b)** hepatic SR-B1 and OPN mRNA levels (n = 21) **(c)** and hepatic SREBP-2 and OPN mRNA levels (n = 21) **(d)**. The correlations were performed using Pearson’s test.

Finally, we validated the correlations between the expression of hepatic OPN and the related hepatic genes in GS. As expected, the mRNA expression of hepatic OPN had a strong positive correlation with the expression of hepatic SHP, ATP8B1, SR-B1 and SREBP-2 in GS (Fig. 6). The results above indicate that hepatic OPN alters the biliary compositions by regulating the expression of the key genes involved in hepatic cholesterol and bile acid metabolism.

## Discussion

In this study, we demonstrated that hepatic OPN is involved in biliary homeostasis by regulating the expression of hepatic key genes, and plays an important role in cholesterol gallstone formation.

Our previous research revealed that OPN can inhibit cholesterol gallstone formation as an anti-nucleation factor in gallbladder bile, and OPN in gallbladder bile is probably from gallbladder tissues^16^,^17^. In the current study, we found that the expression of hepatic OPN was increased in GS, and OPN deficiency altered biliary homeostasis and protected against gallstone formation in mice. It is most likely that hepatic OPN plays a more important role in the process of gallstone formation than gallbladder bile OPN. The increased expression of hepatic OPN in GS confirms that OPN plays a role in the pathogenesis of gallstone disease.

OPN−/− mice showed an increase in body weight than WT mice when fed with LD for 8 weeks. It may be a result of the reduced expression of hepatic β-KLOTHO in LD-fed OPN−/− mice (Fig. 4c), which could result in attenuated hepatic FGF15 signalling^21^. It has been reported that activation of hepatic FGF15 signalling could reduce body weight and promote metabolic rate^22^,^23^,^24^. In addition, we found that the expression of acetyl CoA carboxylase 2 (ACC2)^25^,^26^ and stearoyl CoA desaturase 1 (SCD1)^27^,^28^, which are FGF15 target genes regulating fatty acid oxidation and synthesis, was higher in LD-fed OPN−/− mice than LD-fed WT mice (Supplementary Fig. S2). Thus the increased body weight in LD-fed OPN−/− mice may be partially due to the attenuated hepatic FGF15 signalling, while the detailed mechanism requires further investigation.

It has been reported that male C57BL/6J mice are more susceptible to diet-induced gallstones than female C57BL/6J mice^29^. In our study, equal number of male and female mice were used in two genotypes. And the incidence of gallstone formation in male and female WT mice fed a LD for 8 weeks were the same. Furthermore, the CSI of LD-fed male and female mice showed no significant difference (Supplementary Table S2). These results suggest that the gallstone formation was not affected by the gender of C57BL6/J strain mice under our experiment conditions. By comparing the experiment conditions between our study and the previous study^21^, we found that the LD using in our study contained higher cholesterol contents than the diet (mainly contained 19.4% butter, 3.4% corn oil, 0.38% cholesterol, and 0.5% cholic acid) in previous study^21^. It is likely that under higher cholesterol levels, the influence of gender on diet-induced gallstone disease is reduced.

In the gallbladder bile, the cholesterol solubility is maintained by the balance among cholesterol, bile acid and phospholipid^30^. Thus, LD-fed OPN−/− mice were protected against gallstone formation due to the remarkably lower cholesterol concentration, with higher levels of bile acid and phospholipid in the gallbladder bile and consequently the decreased CSI.

Although the expression of phospholipid efflux ABCB4 was unchanged, the increased biliary phospholipid content in LD-fed OPN−/− mice might be relevant to the reduced expression of ATP8B1, which plays a role in the transport of phospholipid from the outer to the inner canalicular membrane^31^,^32^. Unfortunately, there was no difference in hepatic phospholipid content between the two genotypes.

Regarding the mechanisms of the increased biliary and hepatic bile acid in LD-fed OPN−/− mice, we measured the hepatic genes involved in the metabolism and transportation of bile acid and found that among the genes measured, only the CYP7A1 gene, the rate limiting enzyme of bile acid synthesis^33^, was up-regulated in LD-fed OPN−/− mice. It has been reported that mice overexpressing hepatic CYP7A1 had higher bile acid secretion rates than WT mice^34^. Then we measured the bile acid pool size and the faecal bile acid excretion and found that both were increased in LD-fed OPN−/− mice (Supplementary Fig. S3). In addition, we also examined the expression of intestinal genes involved in bile acid enterohepatic cycling but revealed no statistically significant difference between the genotypes (Supplementary Fig. S4). The increased levels of bile acid pool and faecal bile acid excretion in the setting of none alterations in expression of related intestinal genes suggest that the synthesis and secretion of bile acid in LD-fed OPN−/− mice liver may be increased. However, it is a pity that we did not measure the bile acid secretion rates during the mice experiments, which is a limitation of our study. Taken together, the increase of biliary and hepatic bile acids in LD-fed OPN−/− mice may be mainly due to the up-regulated expression of CYP7A1.

The expression of CYP7A1 is reciprocally regulated by bile acid via the FXR-SHP pathway and by oxysterols via the LXR pathway. LXR combines with retinoid X receptor (RXR) to form a heterodimer. Then, the LXR–RXR heterodimer promotes the expression of CYP7A1 by binding to the promoter region of CYP7A1^33^. Unfortunately, we found no difference in the expression of LXR or RXR. Moreover, it has been shown that the effects of FXR modulating the expression of CYP7A1 are dominant over those of LXR^35^. In the liver, SHP decreases the recruitment of coactivators to the CYP7A1 gene promoter, thus inhibiting the expression of CYP7A1^36^,^37^. Meanwhile, fibroblast growth factor 15 (FGF15) from the intestine could bind to FGFR4 and β-KLOTHO causing a loss of coactivator binding to the CYP7A1 gene promoter and subsequently inhibiting the expression of CYP7A1^38^. We found that the expression of SHP and β-KLOTHO, which is required for the FGF15 signalling^39^,^40^, was decreased in OPN−/− mice fed with LD. These results indicate that the higher expression of CYP7A1 in OPN−/− mice is due to the suppression of SHP. SHP is one target gene of FXR^41^. However, the expression of FXR was not affected in LD-fed OPN−/− mice. These data suggest that loss of OPN results in attenuated SHP signalling. In addition, we confirmed the relevance of our findings in human conditions, observing a strongly positive correlation between the mRNA expression of OPN and SHP in the liver of GS. There are only a few studies of the relationship between OPN and SHP. OPN can induce a significant increase in the level of tumour necrosis factor α (TNFα) in mice^42^. TNFα can also activate the c-Jun N-terminal kinase (JNK) pathway^43^. Moreover, the activation of the JNK pathway can induce SHP expression^44^. These studies suggest that OPN may affect SHP through the TNFα and JNK pathways. Thus, we analysed the hepatic mRNA expression of TNFα in the two mouse genotypes and found that the expression of TNFα was significantly decreased in OPN−/− mice compared with WT mice fed a LD (Supplementary Fig. S5), suggesting that OPN deficiency could suppress the expression of TNFα and results in a reduced expression of SHP. The mechanism by which OPN knockout leads to SHP suppression needs further study.

The biliary cholesterol content was reduced whereas the hepatic cholesterol level was increased in LD-fed OPN−/−mice. This phenomenon may be a result of cholesterol transporter dysfunction. Unexpectedly, the expression of ABCG5 and ABCG8 showed no difference between the genotypes. A previous study suggested that pathways independent of ABCG5 and ABCG8 also exist and contribute to cholesterol secretion into bile^45^. SR-B1 contributes to ABCG5/G8-independent biliary cholesterol secretion, which is localized in the bile canaliculus^10^,^46^. As expected, the expression of SR-B1 mRNA was decreased in OPN−/− mice compared with WT mice. Thus, the main reason for decreased biliary cholesterol profile in LD-fed OPN−/−mice may be the reduced expression of SR-B1. However, SR-B1 also localizes to the hepatocyte membrane as a high density lipoprotein receptor^46^,^47^, taking up cholesterol from serum. Additionally, the LD-fed OPN−/− mice showed an increased hepatic cholesterol level and a decreased expression of the cholesterol synthesis enzyme HMGCR. To further explain this phenomenon, we found that the expression of IDOL and PCSK9, which are SREBP target genes promoting the degradation of LDLR^48^,^49^, were significantly decreased in LD-fed OPN−/− mice, accompanied by the increased protein level of LDLR in LD-fed OPN−/− mice (Supplementary Fig. S6. These results may partially explain the increased hepatic cholesterol level in LD-fed OPN−/− mice.

Unexpectedly, we observed an increased content of hepatic TG in LD-fed OPN−/− mice. We measured the expression of the genes involved in hepatic TG metabolism and found that the mRNA and protein levels of apolipoprotein B (ApoB) were decreased in LD-fed OPN−/− mice (Supplementary Figs S7 and S8). ApoB is used for the constitutive formation of VLDL, which carries both TG and cholesterol in hepatocytes^50^. A previous study showed that the reduction of ApoB lowered the assembly and secretion of VLDL into the circulation, resulting in an increased hepatic TG content^51^. Thus, the increased content of hepatic TG in LD-fed OPN−/− mice may be partially due to the reduced expression of hepatic ApoB.

In conclusion, we have described a previously unknown function of OPN regulating biliary homeostasis, thus affecting the formation of gallstones both in mice and humans. Our results suggest that new therapeutic strategies designed to modulate OPN activity may be beneficial for preventing the formation of cholesterol gallstones.

## Materials and Methods

### Human liver samples

Human liver tissue samples were collected from 21 GS scheduled for laparoscopic cholecystectomy and 14 GSF undergoing other abdominal operations. All gallstone specimens were classified as cholesterol gallstones because all the stones contained greater than 65% cholesterol. No gallstone was found in any of the GSF controls via ultrasonography. None of the patients had shown any clinical or laboratory evidence of diabetes mellitus, hyperlipoproteinaemia, obesity, alcohol abuse, hepatic carcinoma or other conditions that could affect the function of the liver. In addition, none of them took any medications known to affect lipid metabolism. Informed consent was obtained from all participants prior to enrolment in the study, including permission to collect a liver biopsy. The study protocol was approved by the Ethics Committees of Huashan Hospital, Fudan University. The procedures were performed in accordance with the approved guidelines.

### Animals and sample collection

C57BL/6J WT mice were purchased from Fudan University (Shanghai, China). OPN−/− mice in a congenic background were purchased from Jackson Laboratory (Bar Harbor, Maine, USA). WT and OPN−/− mice were fed a CD or LD (CD supplemented with 15% fat, 2% cholesterol, and 0.5% cholic acid) for up to 8 weeks. All experiments were performed on mice between 8 and 10 weeks of age (the gender of the mice is shown in Table 1). All animals received humane care, and their use was approved by the Animal Ethics Committee of Fudan University. All procedures were performed in accordance with the approved guidelines. Bile was collected, and crystal analysis was immediately performed. Blood was collected via right ventricle heart puncture. The gallbladder and liver were harvested and then snap-frozen in liquid nitrogen for protein and RNA isolation or fixed in 4% paraformaldehyde overnight for histological analysis.

### Analysis of lipid and bile acid of bile, plasma, liver and gallstone formation

The hepatic lipid were extracted into chloroform/methanol (2:1)^52^. The bile acid were extracted from liver as described previously^53^. Cholesterol, bile acid and TG levels were measured with assay kits from Kehua Bio-engineering (Shanghai, China), and phospholipid content was measured using an assay kit from Wako (Osaka, Japan) according to the manufacturers’ instructions. All the biochemical measures were assayed in triplicate 3 times. Gallstones were defined as macroscopically visible stones, whereas crystals were defined with polarizing light microscopy (Olympus, Tokyo, Japan). The CSI was calculated according to Carey’s critical tables^54^. These methods were performed in accordance with the approved guidelines from the Animal Ethics Committee of Fudan University.

### Quantitative real-time PCR analysis

Total RNA was obtained from tissue with the RNAprep Pure Tissue kit (TianGen, Beijing, China) according to the manufacturer’s instructions. Random primers (Takara, Shiga, Japan) were used for the reverse transcription of total RNA to complementary DNA. Quantitative real-time PCR was performed using SYBR Green I chemistry (TianGen, Beijing, China) and the ABI 7900HT Fast Real-Time PCR System (Applied Biosystem, Shanghai, China). The gene-specific primer sequences are shown in Supplementary Tables S3 and S4. The mRNA expression levels were calculated relative to the housekeeping gene glyceraldehyde phosphate dehydrogenase (GAPDH) and further normalized to the expression levels of the respective controls following the basis of the relative expression method^55^. These methods were performed in accordance with the approved guidelines from the Animal Ethics Committee of Fudan University.

### Western blot analysis

Frozen liver tissue was lysed in ice-cold RIPA. buffer supplemented with protease inhibitors. The protein concentration of the extracts was measured using the BCA Protein Assay Kit (Thermo, Shanghai, China). The protein was analysed by 8–12% sodium dodecyl sulfate polyacrylamide gel electrophoresis (SDS-PAGE) and transferred to poly (vinylidene fluoride) membranes. Anti-CYP7A1 antibodies (Abcam, Shanghai, China) were used at a 1:1000 dilution, anti-SR-B1 antibodies (Abcam, Shanghai, China) were used at a 1:2000 dilution. A 1:3000 dilution of anti-rabbit immunoglobulin G-HRP (Santa Cruz, Shanghai, China) was used as a secondary antibody. After probing individual antibodies, the antigen-antibody complex was visualized using Enhanced Chemiluminescence Supersignal Reagents (TianGen, Beijing, China). The relative average protein level was determined by densitometry. These methods were performed in accordance with the approved guidelines from the Animal Ethics Committee of Fudan University.

### Immunohistochemistry analysis

Formalin-fixed human biopsies were embedded in paraffin and cut into 3 mm sections. Sample slides were incubated with anti-OPN antibodies (Abcam, Shanghai, China) at a 1:75 dilution, followed by a 30 min incubation with an HRP-labelled polymer secondary antibody (Santa Cruz, Shanghai, China). Sections were viewed with a Nikon ECLIPSE E600 microscope (Nikon, Tokyo, Japan) using 10× objective lenses, and images were acquired with a SPOT INSIGHT™ digital colour camera, model 3.2.0 (Sterling Heights, Michigan, USA). Quantification of immunoreactivity was performed on digitally captured colour images saved as TIFF files and analysed using Image-Pro plus 6.0 (Media Cybernetics, Rockville, Maryland, USA). Blind immunohistochemistry analysis was conducted by a pathologist of Fudan University. These methods were performed in accordance with the approved guidelines from the Animal Ethics Committee of Fudan University.

### Statistical analysis

The data are presented as the mean ± standard deviation (SD) unless otherwise noted. The statistical significance of differences between the means of the experimental groups was evaluated with unpaired Student’s t test, and correlation was performed with Pearson’s test using Prism 6.00 (GraphPad, La Jolla, CA, USA). To meet the criteria of homoscedasticity between variables, the mRNA expression of hepatic OPN was log-transformed, prior to analysis. A difference was considered statistically significant at p < 0.05.

## Additional Information

**How to cite this article**: Lin, J. *et al*. Osteopontin Deficiency Alters Biliary Homeostasis and Protects against Gallstone Formation. *Sci. Rep.* **6**, 30215; doi: 10.1038/srep30215 (2016).

## Supplementary Material

Supplementary Information

## Figures and Tables

**Table 1:** Incidence of cholesterol gallstone formation in WT and OPN−/− mice.

**Diet**	**Genotypes**	**Gallstones**
Chow Diet	**WT**	**0%(0/9)**
	male	0%(0/5)
	female	0%(0/4)
	**OPN−/−**	**0%(0/9)**
	male	0%(0/5)
	female	0%(0/4)
Lithogenic Diet for 8 weeks	**WT**	**80%(8/10)**
	male	80%(4/5)
	female	80%(4/5)
	**OPN−/−**	**10%(1/10)**
	male	20%(1/5)
	female	0%(0/5)

The data are expressed as the percentage.

WT, wild type; OPN−/−, OPN deficient.

**Table 2:** Body weight, liver weight, serum lipids and hepatic lipids of WT and OPN−/− mice.

**Parameters**	**Chow Diet**		**Lithogenic Diet**	
	**WT**	**OPN**−/−	**WT**	**OPN**−/−
Body weight (g)	20.1 ± 1.06	21.2 ± 1.47	20.1 ± 0.80	23.9 ± 2.90**
Liver weight (% body weight)	4.90 ± 0.31	4.54 ± 0.39	4.59 ± 0.24	4.33 ± 0.20*
Hepatic cholesterol (mg/g liver)	2.04 ± 0.33	1.91 ± 0.41	3.44 ± 0.97	4.64 ± 0.82**
Hepatic TG (mg/g liver)	8.15 ± 3.09	7.41 ± 2.05	9.63 ± 1.98	13.5 ± 4.00*
Hepatic phospholipid (mg/g liver)	10.6 ± 2.34	12.4 ± 1.96	15.2 ± 1.67	19.0 ± 4.61
Hepatic bile acid (μmol/g liver)	0.51 ± 0.26	0.57 ± 0.24	0.75 ± 0.19	1.14 ± 0.36**
Serum cholesterol (mmol/L)	1.84 ± 0.21	1.96 ± 0.28	2.68 ± 0.90	2.37 ± 0.52
Serum TG (mmol/L)	0.35 ± 0.17	0.38 ± 0.17	0.46 ± 0.73	0.29 ± 0.03
Serum phospholipid (mmol/L)	1.97 ± 0.22	2.07 ± 0.38	2.09 ± 0.75	2.41 ± 0.29
Serum bile acid (μmol/L)	4.54 ± 4.27	3.82 ± 2.79	10.3 ± 8.03	18.17 ± 10.4

The data are expressed as the mean ± SD (n = 8 per group). Statistical analysis was performed using unpaired Student’s t test, *P < 0.05, **P < 0.01.

WT, wild type; OPN−/−, OPN deficient; TG, triglyceride.

## References

[b1] Sandler R. S. . The burden of selected digestive diseases in the United States. Gastroenterology 122, 1500–1511 (2002). 11984534 10.1053/gast.2002.32978

[b2] Everhart J. E. & Ruhl C. E. Burden of digestive diseases in the United States Part III: Liver, biliary tract, and pancreas. Gastroenterology 136, 1134–1144 (2009). 19245868 10.1053/j.gastro.2009.02.038

[b3] Kaechele V. . Prevalence of gallbladder stone disease in obese children and adolescents: influence of the degree of obesity, sex, and pubertal development. J Pediatr Gastroenterol Nutr 42, 66–70 (2006). 16385256 10.1097/01.mpg.0000187816.31213.06

[b4] Shaffer E. A. Gallstone disease: Epidemiology of gallbladder stone disease. Best Pract Res Clin Gastroenterol 20, 981–996 (2006). 17127183 10.1016/j.bpg.2006.05.004

[b5] Portincasa P., Moschetta A. & Palasciano G. Cholesterol gallstone disease. Lancet 368, 230–239 (2006). 16844493 10.1016/S0140-6736(06)69044-2

[b6] Lamont J. T. & Carey M. C. Cholesterol gallstone formation. 2. Pathobiology and pathomechanics. Prog Liver Dis 10, 165–191 (1992). 1296229

[b7] Hofmann A. F., Amelsberg A. & VanSonnenberg E. Pathogenesis and treatment of gallstones. N Engl J Med 328, 1854–1855 (1993). 8502282

[b8] Busch N. & Matern S. Current concepts in cholesterol gallstone pathogenesis. Eur J Clin Invest 21, 453–460 (1991). 1752283 10.1111/j.1365-2362.1991.tb01394.x

[b9] Dikkers A., Freak D. B. J., Annema W., Groen A. K. & Tietge U. J. Scavenger receptor BI and ABCG5/G8 differentially impact biliary sterol secretion and reverse cholesterol transport in mice. Hepatology 58, 293–303 (2013). 23401258 10.1002/hep.26316

[b10] Wiersma H. . Scavenger receptor class B type I mediates biliary cholesterol secretion independent of ATP-binding cassette transporter g5/g8 in mice. Hepatology 50, 1263–1272 (2009). 19637290 10.1002/hep.23112

[b11] Yu L. . Expression of ABCG5 and ABCG8 is required for regulation of biliary cholesterol secretion. J Biol Chem 280, 8742–8747 (2005). 15611112 10.1074/jbc.M411080200

[b12] Smit J. J. . Homozygous disruption of the murine mdr2 P-glycoprotein gene leads to a complete absence of phospholipid from bile and to liver disease. Cell 75, 451–462 (1993). 8106172 10.1016/0092-8674(93)90380-9

[b13] Dawson P. A., Lan T. & Rao A. Bile acid transporters. J Lipid Res 50, 2340–2357 (2009). 19498215 10.1194/jlr.R900012-JLR200 PMC2781307

[b14] Wang K. X. & Denhardt D. T. Osteopontin: role in immune regulation and stress responses. Cytokine Growth Factor Rev 19, 333–345 (2008). 18952487 10.1016/j.cytogfr.2008.08.001

[b15] Rangaswami H., Bulbule A. & Kundu G. C. Osteopontin: role in cell signaling and cancer progression. Trends Cell Biol 16, 79–87 (2006). 16406521 10.1016/j.tcb.2005.12.005

[b16] Yang L., Chen J. H., Cai D., Wang L. Y. & Zha X. L. Osteopontin and integrin are involved in cholesterol gallstone formation. Med Sci Monit 18, R16–R23 (2012). 10.12659/MSM.882194 PMC3560682 22207105

[b17] Yang L., Chen J. H., Cai D., Wang L. Y. & Zha X. L. Osteopontin plays an anti-nucleation role in cholesterol gallstone formation. Hepatol Res 41, 437–445 (2011). 21435127 10.1111/j.1872-034X.2011.00790.x

[b18] Nakai A., Imano M., Takeyama Y., Shiozaki H. & Ohyanagi H. An immunohistochemical study of osteopontin in hepatolithiasis. J Hepatobiliary Pancreat Surg 15, 615–621 (2008). 18987932 10.1007/s00534-007-1320-8

[b19] Chapman J. . Osteopontin is required for the early onset of high fat diet-induced insulin resistance in mice. Plos One 5, e13959 (2010). 21103061 10.1371/journal.pone.0013959 PMC2980483

[b20] Biddinger S. B. . Hepatic insulin resistance directly promotes formation of cholesterol gallstones. Nat Med 14, 778–782 (2008). 18587407 10.1038/nm1785 PMC2753607

[b21] Wu X. . Co-receptor requirements for fibroblast growth factor-19 signaling. J Biol Chem 282, 29069–29072 (2007). 17711860 10.1074/jbc.C700130200

[b22] Fu L. . Fibroblast growth factor 19 increases metabolic rate and reverses dietary and leptin-deficient diabetes. Endocrinology 145, 2594–2603 (2004). 14976145 10.1210/en.2003-1671

[b23] Tomlinson E. . Transgenic mice expressing human fibroblast growth factor-19 display increased metabolic rate and decreased adiposity. Endocrinology 143, 1741–1747 (2002). 11956156 10.1210/endo.143.5.8850

[b24] Rysz J., Gluba-Brzozka A., Mikhailidis D. P. & Banach M. Fibroblast growth factor 19-targeted therapies for the treatment of metabolic disease. Expert Opin Investig Drugs 24, 603–610 (2015). 10.1517/13543784.2015.1006357 25604607

[b25] Abu-Elheiga L., Matzuk M. M., Abo-Hashema K. A. & Wakil S. J. Continuous fatty acid oxidation and reduced fat storage in mice lacking acetyl-CoA carboxylase 2. Science 291, 2613–2616 (2001). 11283375 10.1126/science.1056843

[b26] Abu-Elheiga L., Oh W., Kordari P. & Wakil S. J. Acetyl-CoA carboxylase 2 mutant mice are protected against obesity and diabetes induced by high-fat/high-carbohydrate diets. Proc Natl Acad Sci USA 100, 10207–10212 (2003). 12920182 10.1073/pnas.1733877100 PMC193540

[b27] Cohen P. . Role for stearoyl-CoA desaturase-1 in leptin-mediated weight loss. Science 297, 240–243 (2002). 12114623 10.1126/science.1071527

[b28] Flowers M. T. & Ntambi J. M. Role of stearoyl-coenzyme A desaturase in regulating lipid metabolism. Curr Opin Lipidol 19, 248–256 (2008). 18460915 10.1097/MOL.0b013e3282f9b54d PMC4201499

[b29] Alexander M. & Portman O. W. Different susceptibilities to the formation of cholesterol gallstones in mice. Hepatology 7, 257–265 (1987). 3557305 10.1002/hep.1840070209

[b30] Wang D. Q. & Carey M. C. Complete mapping of crystallization pathways during cholesterol precipitation from model bile: influence of physical-chemical variables of pathophysiologic relevance and identification of a stable liquid crystalline state in cold, dilute and hydrophilic bile salt-containing systems. J Lipid Res 37, 606–630 (1996). 8728323

[b31] Paulusma C. C. . ATP8B1 requires an accessory protein for endoplasmic reticulum exit and plasma membrane lipid flippase activity. Hepatology 47, 268–278 (2008). 17948906 10.1002/hep.21950

[b32] Folmer D. E., Elferink R. P. & Paulusma C. C. P4 ATPases-lipid flippases and their role in disease. Biochim Biophys Acta 1791, 628–635 (2009). 19254779 10.1016/j.bbalip.2009.02.008

[b33] Russell D. W. The enzymes, regulation, and genetics of bile acid synthesis. Annu Rev Biochem 72, 137–174 (2003). 12543708 10.1146/annurev.biochem.72.121801.161712

[b34] Li T. . Overexpression of cholesterol 7alpha-hydroxylase promotes hepatic bile acid synthesis and secretion and maintains cholesterol homeostasis. Hepatology 53, 996–1006 (2011). 21319191 10.1002/hep.24107 PMC3079544

[b35] Ando H. . Regulation of cholesterol 7alpha-hydroxylase mRNA expression in C57BL/6 mice fed an atherogenic diet. Atherosclerosis 178, 265–269 (2005). 15694933 10.1016/j.atherosclerosis.2004.09.016

[b36] Goodwin B. . A regulatory cascade of the nuclear receptors FXR, SHP-1, and LRH-1 represses bile acid biosynthesis. Mol Cell 6, 517–526 (2000). 11030332 10.1016/s1097-2765(00)00051-4

[b37] Lu T. T. . Molecular basis for feedback regulation of bile acid synthesis by nuclear receptors. Mol Cell 6, 507–515 (2000). 11030331 10.1016/s1097-2765(00)00050-2

[b38] Inagaki T. . Fibroblast growth factor 15 functions as an enterohepatic signal to regulate bile acid homeostasis. Cell Metab 2, 217–225 (2005). 16213224 10.1016/j.cmet.2005.09.001

[b39] Wu X. . Co-receptor requirements for fibroblast growth factor-19 signaling. J Biol Chem 282, 29069–29072 (2007). 17711860 10.1074/jbc.C700130200

[b40] Moore L. B.. St. John’s wort induces hepatic drug metabolism through activation of the pregnane X receptor. Proc Natl Acad Sci USA 97, 7500–7502 (2000). 10852961 10.1073/pnas.130155097 PMC16574

[b41] Bertolotti M. . Nuclear receptors as potential molecular targets in cholesterol accumulation conditions: insights from evidence on hepatic cholesterol degradation and gallstone disease in humans. Curr Med Chem 15, 2271–2284 (2008). 18781949 10.2174/092986708785747544

[b42] Zhu Y. . Osteopontin Exacerbates Pulmonary Damage in Influenza-Induced Lung Injury. Jpn J Infect Dis 68, 467–473 (2015). 25866117 10.7883/yoken.JJID.2014.467

[b43] Weston C. R. & Davis R. J. The JNK signal transduction pathway. Curr Opin Genet Dev 12, 14–21 (2002). 11790549 10.1016/s0959-437x(01)00258-1

[b44] Gupta S., Stravitz R. T., Dent P. & Hylemon P. B. Down-regulation of cholesterol 7alpha-hydroxylase (CYP7A1) gene expression by bile acids in primary rat hepatocytes is mediated by the c-Jun N-terminal kinase pathway. J Biol Chem 276, 15816–15822 (2001). 11278771 10.1074/jbc.M010878200

[b45] Geuken E. . Hepatic expression of ABC transporters G5 and G8 does not correlate with biliary cholesterol secretion in liver transplant patients. Hepatology 42, 1166–1174 (2005). 16250035 10.1002/hep.20886

[b46] Acton S. . Identification of scavenger receptor SR-BI as a high density lipoprotein receptor. Science 271, 518–520 (1996). 8560269 10.1126/science.271.5248.518

[b47] Kozarsky K. F. . Overexpression of the HDL receptor SR-BI alters plasma HDL and bile cholesterol levels. Nature 387, 414–417 (1997). 9163428 10.1038/387414a0

[b48] Zelcer N., Hong C., Boyadjian R. & Tontonoz P. LXR regulates cholesterol uptake through Idol-dependent ubiquitination of the LDL receptor. Science 325, 100–104 (2009). 19520913 10.1126/science.1168974 PMC2777523

[b49] Bergeron N., Phan B. A., Ding Y., Fong A. & Krauss R. M. Proprotein convertase subtilisin/kexin type 9 inhibition: a new therapeutic mechanism for reducing cardiovascular disease risk. Circulation 132, 1648–1666 (2015). 26503748 10.1161/CIRCULATIONAHA.115.016080

[b50] Morita S. Y. Metabolism and Modification of Apolipoprotein B-Containing Lipoproteins Involved in Dyslipidemia and Atherosclerosis. Biol Pharm Bull 39, 1–24 (2016). 26725424 10.1248/bpb.b15-00716

[b51] Reyes-Soffer G. . Complex effects of inhibiting hepatic apolipoprotein B100 synthesis in humans. Sci Transl Med 8, 312r–323r (2016). 10.1126/scitranslmed.aad2195 PMC4944115 26819195

[b52] Nervi F., Marinovic I., Rigotti A. & Ulloa N. Regulation of biliary cholesterol secretion. Functional relationship between the canalicular and sinusoidal cholesterol secretory pathways in the rat. J Clin Invest 82, 1818–1825 (1988). 3198756 10.1172/JCI113797 PMC442759

[b53] Csanaky I. L. . Organic anion-transporting polypeptide 1b2 (Oatp1b2) is important for the hepatic uptake of unconjugated bile acids: Studies in Oatp1b2-null mice. Hepatology 53, 272–281 (2011). 20949553 10.1002/hep.23984 PMC3186067

[b54] Carey M. C. Critical tables for calculating the cholesterol saturation of native bile. J Lipid Res 19, 945–955 (1978). 731129

[b55] Livak K. J. & Schmittgen T. D. Analysis of relative gene expression data using real-time quantitative PCR and the 2(-Delta Delta C(T)) Method. Methods 25, 402–408 (2001). 11846609 10.1006/meth.2001.1262

