# Erratum: Osteopontin Deficiency Alters Biliary Homeostasis and Protects against Gallstone Formation

**DOI:** 10.1038/srep44962

**Published:** 2017-03-22

**Authors:** Jing Lin, Ming Lu, Wei-qing Shao, Zong-you Chen, Wen-wei Zhu, Lu Lu, Hu-liang Jia, Duan Cai, Lun-xiu Qin, Jin-hong Chen

Scientific Reports 6: Article number: 30215; 10.1038/srep30215 published online: 08 03 2016; updated: 03 22 2017.

The original version of this Article contained an error in the order of author names, which were incorrectly given as ‘Jing Lin, Wei-qing Shao, Zong-you Chen, Wen-wei Zhu, Lu Lu, Duan Cai, Lun-xiu Qin, Hu-liang Jia, Ming Lu & Jin-hong Chen’.

These errors have now been corrected in the PDF and HTML versions of the Article.

