# Supplementary material for: Osteopontin Deficiency Alters Biliary Homeostasis and Protects against Gallstone Formation
Source: Sci Rep. 2016 Aug 3;6:30215. doi: 10.1038/srep30215 (PMC4971489; doi:10.1038/srep30215)
Supplement: Supplementary Information [file srep30215-s1.pdf]

Supplementary information

Osteopontin Deficiency Alters Biliary Homeostasis and Protects against Gallstone Formation

Jing Lin*, Wei-qing Shao*, Zong-you Chen, Wen-wei Zhu, Lu Lu, Duan Cai, Lun-xiu Qin, Hu-liang Jia, Ming Lu and Jin-hong Chen†

Department of General Surgery, Huashan Hospital, Fudan University, Shanghai, 200040, China

* These authors contributed equally to this work.

† Correspondence to: J.-h. C. (email: jinhongch@hotmail.com)

Supplementary Materials and Methods

Histopathological analysis

Mouse tissues were fixed in 4% paraformaldehyde overnight and transferred to 70% ethanol prior to processing and staining with haematoxylin and eosin. Sections were viewed with a Nikon ECLIPSE E600 microscope (Nikon, Tokyo, Japan) using 10× objective lenses, and images were acquired with a SPOT INSIGHT™ digital colour camera, model 3.2.0 (Sterling Heights, Michigan, USA). Blind histopathological analysis was conducted by a pathologist of Fudan University. These methods were performed in accordance with the approved guidelines from the Animal Ethics Committee of Fudan University.

Analysis of bile acid pool size and faecal bile acid excretion

Bile acid pool size was expressed as the sum total bile acid contents of the entire small intestine, gallbladder, and liver, as described previously[1]. In addition, faecal bile acid excretion was determined from stool quantitatively collected from individually housed mice for periods up to 72 hours, as described previously[1, 2]. Then, the bile acid content was measured enzymatically as described. These methods were performed in accordance with the approved guidelines from the Animal Ethics Committee of Fudan University.

References:

1. Schwarz, M., Russell, D.W., Dietschy, J.M. & Turley, S.D. Marked reduction in bile acid synthesis in cholesterol 7alpha-hydroxylase-deficient mice does not lead to diminished tissue cholesterol turnover or to hypercholesterolemia. J Lipid Res 39, 1833-43 (1998).
2. Setchell, K.D., Lawson, A.M., Tanida, N. & Sjovall, J. General methods for the analysis of metabolic profiles of bile acids and related compounds in feces. J Lipid Res 24, 1085-100 (1983).

Supplementary Figures

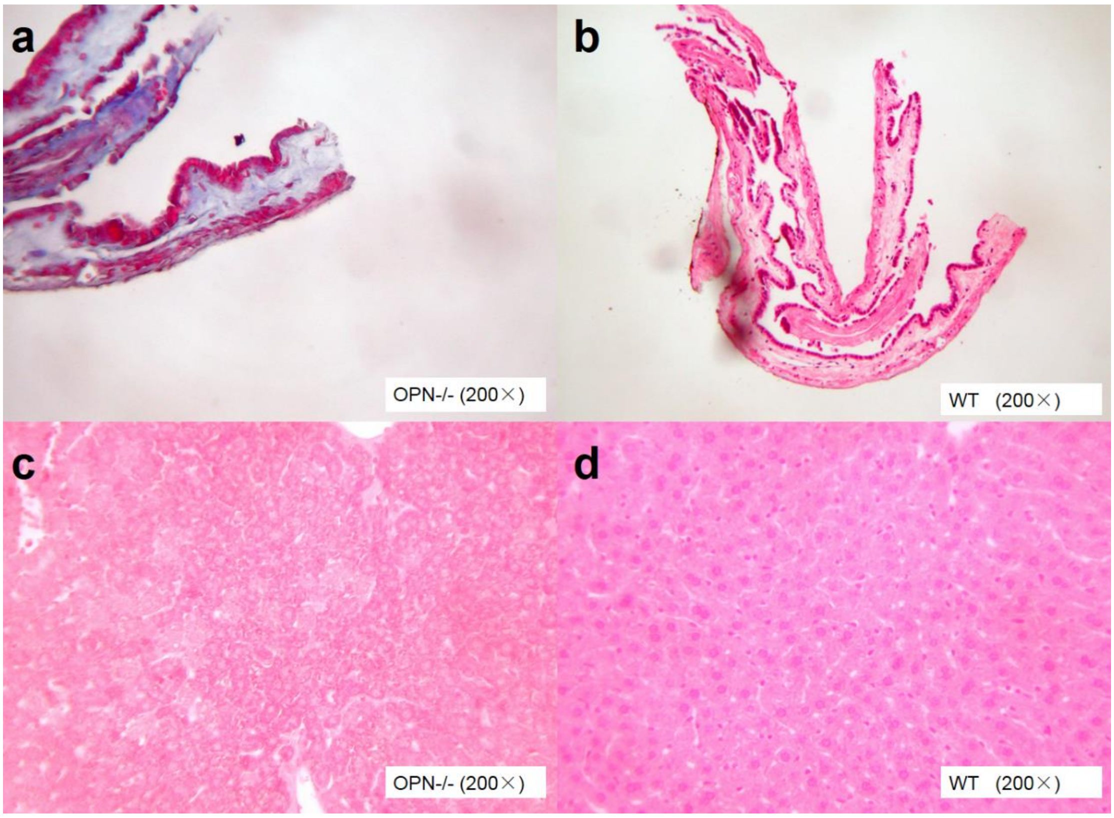

Supplementary Figure S1. Histological examination of the gallbladder and liver in mice fed a lithogenic diet by H&E staining.

(a, b) Histological examination of the gallbladder by H&E staining (magnification, ×200). **(c, d)** Histological examination of the liver by H&E staining (magnification, ×200).

WT, wild type; OPN-/-, OPN deficient.

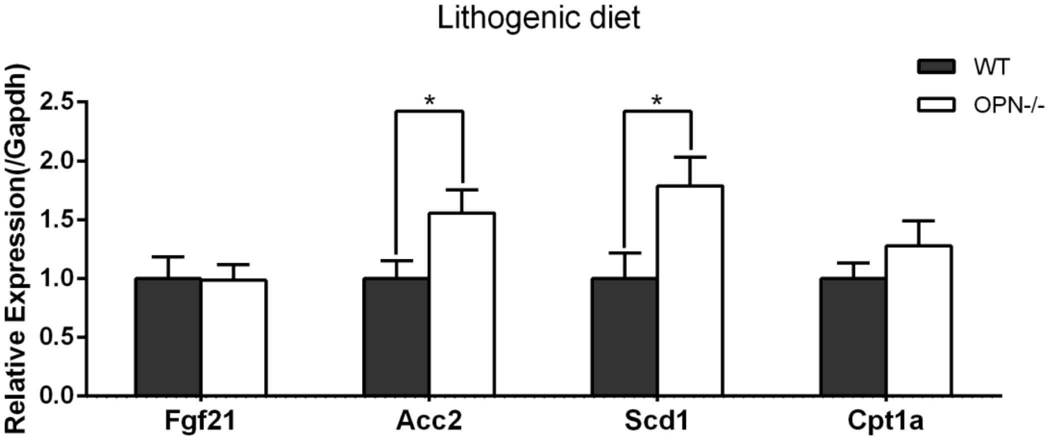

Supplementary Figure S2. Expression of FGF15-associated hepatic genes in mice fed a lithogenic diet.

Quantitative real time-PCR analysis of mRNA levels of FGF 15-associated hepatic genes in mice fed a lithogenic diet for 8 weeks. The data are expressed as the mean ± SD (n=8 per group). Statistical analysis was performed using unpaired Student's t test, * P<0.05, **P<0.01.

WT, wild type; OPN-/-, OPN deficient.

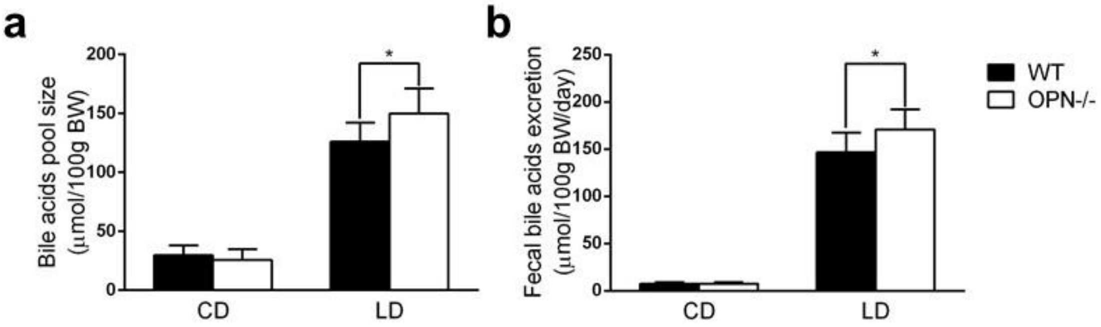

Supplementary Figure S3. The bile acid pool size and faecal bile acid excretion in mice.

The bile acid pool size **(a)** and faecal bile acid excretions **(b)** were measured in OPN-/- mice and WT mice fed a CD or LD. The data are expressed as the mean ± SD (n=8 per group). Statistical analysis was performed using unpaired Student's t test, * P<0.05, **P<0.01.

WT, wild type; OPN-/-, OPN deficient; CD, chow diet, LD, lithogenic diet.

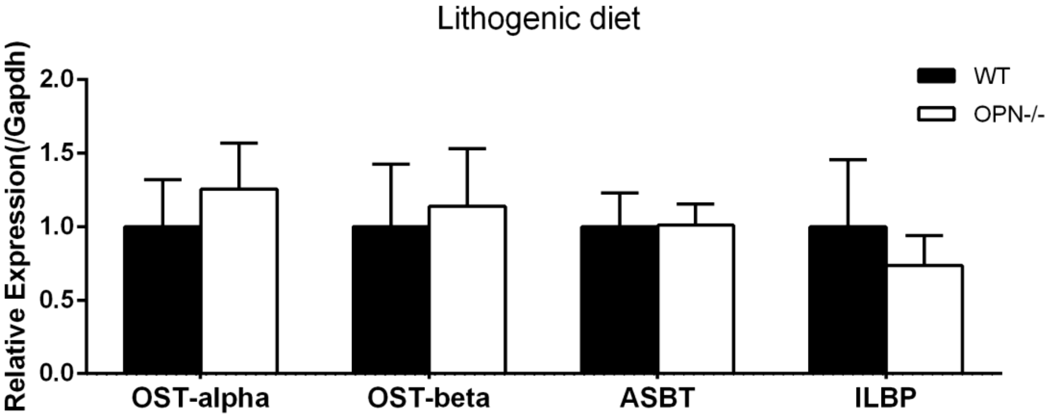

Supplementary Figure S4. Expression of bile acid metabolism-related intestinal genes in mice fed a lithogenic diet.

Quantitative real time-PCR analysis of mRNA levels of intestinal genes involved in bile acid metabolism in mice fed a lithogenic diet for 8 weeks. The data are expressed as the mean ± SD (n=8 per group). Statistical analysis was performed using unpaired Student's t test, * $P<0.05$, ** $P<0.01$.

WT, wild type; OPN-/-, OPN deficient.

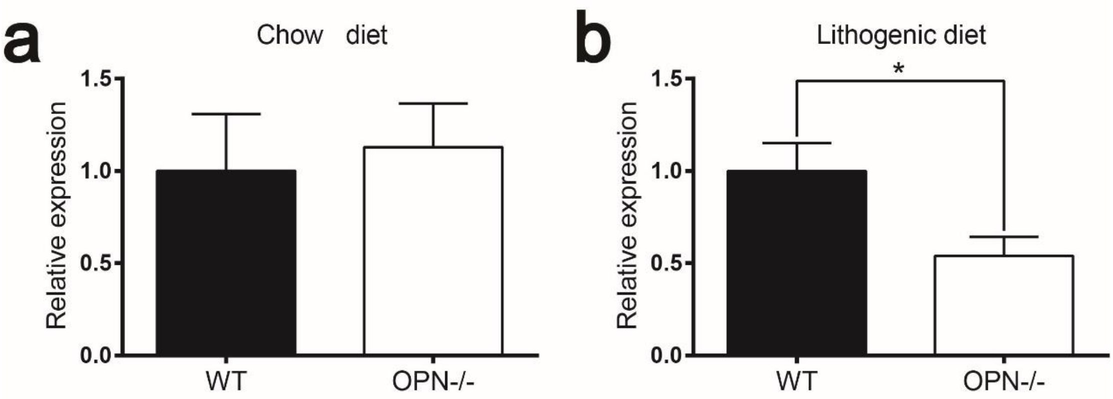

Supplementary Figure S5. Hepatic mRNA expression of TNFα in mice by quantitative real-time PCR.

Quantitative real-time PCR analysis of mRNA levels of TNFα in liver tissues from WT mice and OPN-/- mice fed a chow diet **(a)** and lithogenic diet **(b)**. The data are expressed as the mean ± SD (n=8 per group). Statistical analysis was performed using unpaired Student's t test, * $P<0.05$, ** $P<0.01$.

WT, wild type; OPN-/-, OPN deficient.

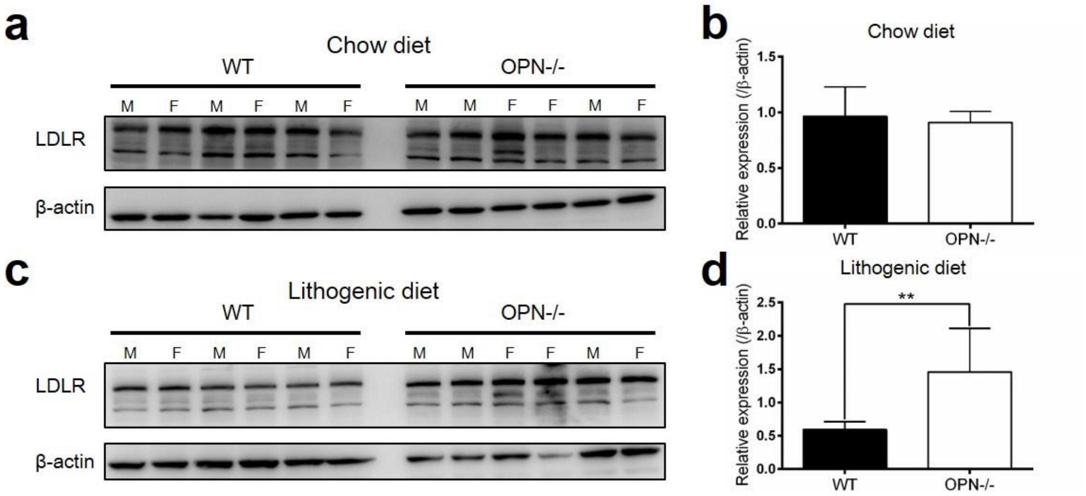

Supplementary Figure S6. Western blot analysis of the protein expression of LDLR in mice.

(a,c) Hepatic proteins were isolated from WT and OPN-/- mice and analysed by Western blotting for LDLR protein expression. The gender of mice was labelled above the bands. β-Actin was selected as a control for gel loading. Anti-LDLR antibodies (Cayman, Michigan, USA) were used at a 1:500 dilution. A 1:3000 dilution of anti-rabbit immunoglobulin G-HRP (Santa Cruz, Shanghai, China) was used as a secondary antibody. **(b, d)** Quantification of Western blots. The relative average protein level was determined by densitometry. The data are expressed as the mean ± SD (n=6 per group, 3 female and 3 male mice). Statistical analysis was performed using unpaired Student's t test, * $P<0.05$, ** $P<0.01$.

WT, wild type; OPN-/-, OPN deficient; M, male; F, female.

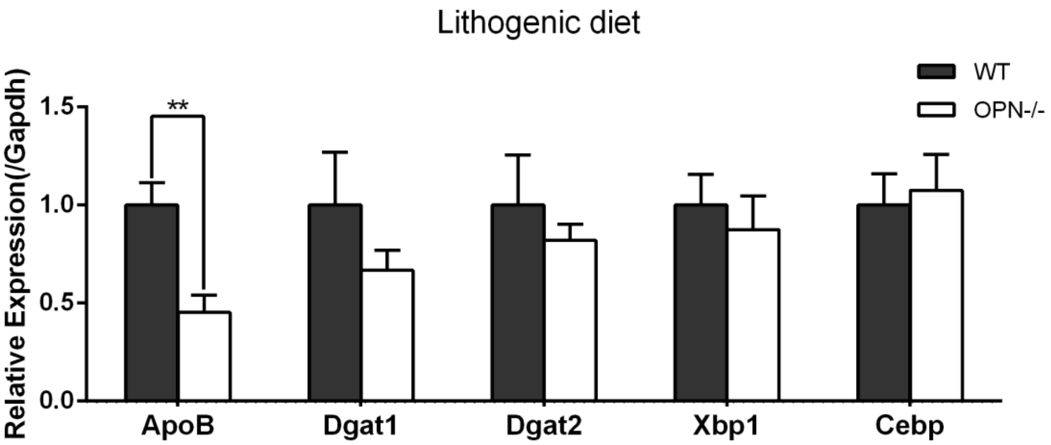

Supplementary Figure S7. Expression of TG metabolism-related genes in mice fed a lithogenic diet.

Quantitative real-time PCR analysis of mRNA levels for genes involved in TG metabolism in liver tissues from WT mice and OPN-/- mice fed a lithogenic diet. The data are expressed as the mean ± SD (n=8 per group). Statistical analysis was performed using unpaired Student's t test, * P<0.05, **P<0.01.

WT, wild type; OPN-/-, OPN deficient; TG, triglyceride.

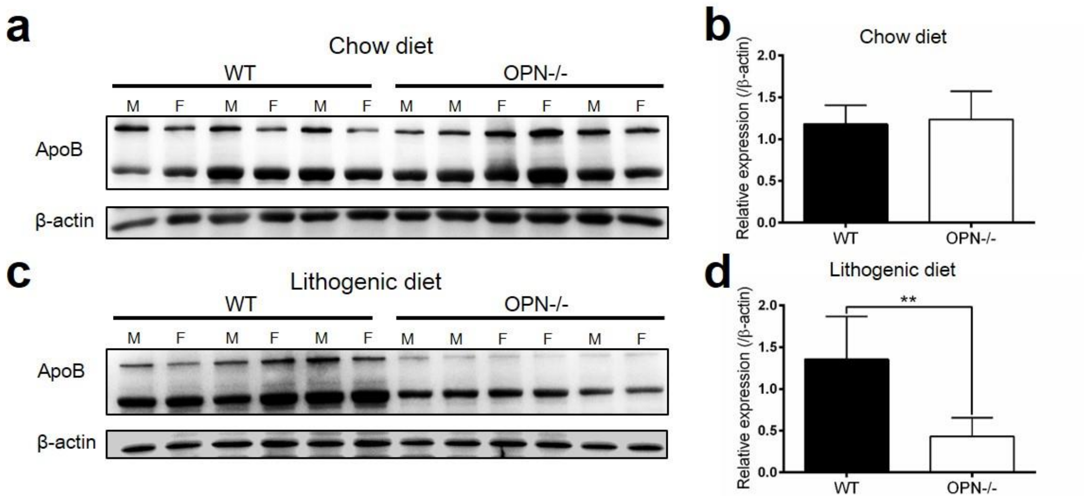

Supplementary Figure S8. Western blot analysis of the protein expression of ApoB in mice.

(a,c) Hepatic proteins were isolated from WT and OPN-/- mice and analysed by Western blotting for ApoB protein expression. The gender of mice was labelled above the bands. β-Actin was selected as a control for gel loading. Anti- ApoB antibodies (Abcam, Shanghai, China) were used at a 1:500 dilution. A 1:3000 dilution of anti-rabbit immunoglobulin G-HRP (Santa Cruz, Shanghai, China) was used as a secondary antibody. **(b, d)** Quantification of Western blots. The relative average protein level was determined by densitometry. The data are expressed as the mean ± SD (n=6 per group, 3 female and 3 male mice). Statistical analysis was performed using unpaired Student's t test, * P<0.05, **P<0.01.

WT, wild type; OPN-/-, OPN deficient; M, male; F, female.

Supplementary Tables

Supplementary Table S1. Age, gender, body mass index and fasting glucose of GS and GSF.

Variable	GS (n=21)	GSF (n=14)	P value	Statistical analysis
Age (years)	56.29±10.49	47.07±18.78	0.151	Wilcoxon test
Gender (female/male)	15/6	8/6	0.477	Fisher exact test
Body mass index (kg/m^2)	22.96±3.03	22.27±3.88	0.555	Student's t test
Fasting glucose (mmol/L)	5.22±0.92	4.81±0.55	0.303	Wilcoxon test

The data are expressed as the mean ± SD (GS: n=21, GSF: n=14).

GS, gallstone patients; GSF, gallstone-free patients.

Supplementary Table S2. The CSI of mice fed a lithogenic diet for 8 weeks.

Genotypes	Male	Female	P values (male *vs* female)
OPN-/-	0.64±0.10	0.55±0.14	0.30
WT	1.33±0.23	1.11±0.18	0.13

The data are expressed as the mean ± SD (n=5 per group). Statistical analysis was performed using unpaired Student's t test.

WT, wild type; OPN-/-, OPN deficient; CSI, cholesterol saturation index.

Supplementary Table S3. Primer sequences for quantitative real-time PCR in mice.

Gene	Sense Primer (5´→3´)	Antisense Primer (5´→3´)
LXRα	GATAGGGTTGGAGTCAGCA	GGAGCGCCTGTTACACTGTT
LXRβ	TGCAACAAACGATCTTTCTCC	TCTCGGGACTGAGGGTCTG
CYP7A1	AGCAACTAAACAACCTGCCAGTACTA	GTCCGGATATTCAAGGATGCA
CYP27A1	GATCTTCATCGCACAAGGAG	GATAACCTCGTTTAAGGCATCC
CYP7B1	GGAGCCACGACCCTAGATG	TGCCAAGATAAGGAAGCCAAC
CYP8B1	CCTCTGGACAAGGGTTTTGTG	GCACCGTGAAGACATCCCC
ABCB1a	CAGCAGTCAGTGTGCTTACAA	ATGGCTCTTTTATCGGCCTCA
ABCB1b	CTGTTGGCGTATTTGGGATGT	CAGCATCAAGAGGGGAAGTAATG
ABCB11	TCTGACTCAGTGATTCTTCGCA	CCCATAAACATCAGCCAGTTGT
ATP8B1	ACGGAAGACACTACTATCTGCT	CACGGTTTTCTCCAGGTGG
ABCB4	GGCTGAGATGGATCTTGAG	CCTTGGTTGCTGATGCTG
LDLR	TGGCTGTTCCCACATCTG	CTCGTCAATATCTTCACACCTG
LRP	ACTATGGATGCCCCTAAAACTTG	GCAATCTCTTTCACCGTCACA
SR-B1	CTCCCAGACATGCTTCCCA	CCGTTCCATTTGTCCACCA
ABCG5	AGGGCCTCACATCAACAGAG	GCTGACGCTGTAGGACACAT
ABCG8	TGCCCACCTTCCACATGTC	ATGAAGCCGGCAGTAAGGTAGA
NPC1	TCTCTGAGACCTCGGCATTT	CAGGATGTCCAGACGGTTTT
NPC2	AGAGCTTTCATATCCACGA	TTAGAGCCGCAGTCCT

SCP2	CCTTCTGTCGCTTTGAAATCTCC	GCTTCCTTTGCCATATCAGGAT
FXR	AGCGAAGGGCGTGAC	TTCCGTTTTCTCCCTGCAAA
SHP	GGAGGCCTTGGATGTCCTAG	AGCCTCCTGTTGCAGGTGTG
FGFR4	AGTCAAATGGATGGCTCCAG	GAGGGTGAAGATTTCCCACA
β-KLOTHO	TCCCCTGTGATTTCTCTTGG	GAGCAATCTGTTGCCAGTGA
HMGCR	CTTGTGGAATGCCTTGTGATTG	AGCCGAAGCAGCACATGAT
HMGCS	GCCGTGAACTGGGTCGAA	GCATATATAGCAATGTCTCCTGCAA
SREBP1c	GGAGCCATGGATTGCACATT	GGCCCGGGAAGTCACTGT
SREBP2	GCGTTCTGGAGACCATGGA	ACAAAGTTGCTCTGAAAACAAATCA
OATP4	CCTGAGAAGTGTCCGCATAAC	GTCCGAGTGGCAGTAAGAAAG
ABCA1	TCCTGGTGTGGAAAAATTGGC	GTGCGAGGTAACAAGTTGGTT
NTCP	CAAACCTCAGAAGGACCAAACA	GTAGGAGGATTATTCCCGTTGTG
PXR	GATGGAGGTCTTCAAATCTGCC	GGCCCTTCTGAAAAACCCCT
PPARα	AGAGCCCCATCTGTCCTCTC	ACTGGTAGTCTGCAAAACCAAA
RXRα	ATGGACACCAAACATTTCCTGC	CCAGTGGAGAGCCGATTCC
TNFα	CCCTCACACTCAGATCATCTTCT	GCTACGACGTGGGCTACAG
PCSK9	ACCCTCATAGGCCTGGAGTT	CTGTGATGACCTCTGGAGCA
IDOL	ATGCTGTGCTATGTGACGAGG	TCGATGATCCCTAGACGCCTG
APOB	AAGCACCTCCGAAAGTACGTG	CTCCAGCTCTACCTTACAGTTGA
DGAT1	TCCGTCCAGGGTGGTAGTG	TGAACAAAGAATCTTGCAGACGA
DGAT2	GCGCTACTTCCGAGACTACTT	GGGCCTTATGCCAGGAAACT
XBP1	AGCAGCAAGTGGTGGATTTG	GAGTTTTCTCCCGTAAAAGCTGA

CEBP	CAAGAACAGCAACGAGTACCG	GTCACTGGTCAACTCCAGCAC
OSTα	AGGCAGGACTCATATCAAACTTG	TGAGGGCTATGTCCACTGGG
OSTβ	AGATGCGGCTCCTTGGAATTA	TGGCTGCTTCTTTCGATTTCTG
ASBT	GTCTGTCCCCCAAATGCAACT	CACCCCATAGAAAACATCACCA
ILBP	CTTCCAGGAGACGTGATTGAAA	CCTCCGAAGTCTGGTGATAGTTG
FGF21	CTGCTGGGGGTCTACCAAG	CTGCGCCTACCACTGTTCC
ACC2	CGCTCACCAACAGTAAGGTGG	GCTTGGCAGGGAGTTCCTC
SCD1	TTCTTGCGATACACTCTGGTGC	CGGGATTGAATGTTCTTGTCGT
CPT1a	CTCCGCCTGAGCCATGAAG	CACCAGTGATGATGCCATTCT
GAPDH	TGTGTCCGTCGTGGATCTGA	CCTGCTTCACCACCTTCTTGAT

Supplementary Table S4. Primer sequences for quantitative real-time PCR in human.

Gene	Sense Primer (5´→3´)	Antisense Primer (5´→3´)
OPN	CTGGTGCTCGTCCTCTACTAC	GGACACGAAGGTAAAGGTGAC
SHP	AGAATATGCCTGCCTGAA	TGGTCGGAATGGACTTGA
ATP8B1	GCAATTTGGTGCCAATCCTCT	GCACAACACCTTATGGTATGACA
SRB1	GCTCGGAGAGCGACTACATC	CCACATGATCTCACCCACAG
SREBP2	CCTGGGAGACATCGACGAGAT	TGAATGACCGTTGCACTGAAG
GAPDH	ACCCACTCCTCCACCTTTG	CTGTAGCCAAATTCGTTGTCAT
